# A case of neoadjuvant chemotherapy‐resistant muscle‐invasive bladder cancer that markedly responded to pembrolizumab before conversion radical cystectomy

**DOI:** 10.1002/iju5.12669

**Published:** 2023-11-12

**Authors:** Ichiro Yonese, Noboru Numao, Kentaro Inamura, Yusuke Yoneoka, Ryo Fujiwara, Yosuke Yasuda, Tomohiko Oguchi, Shinya Yamamoto, Takeshi Yuasa, Junji Yonese

**Affiliations:** ^1^ Department of Urology, Cancer Institute Hospital Japanese Foundation for Cancer Research Tokyo Japan; ^2^ Department of Pathology, Cancer Institute Hospital Japanese Foundation for Cancer Research Tokyo Japan

**Keywords:** bladder cancer, conversion surgery, neoadjuvant chemotherapy, neoadjuvant immunotherapy

## Abstract

**Introduction:**

Recently, perioperative use of immune checkpoint inhibitors has improved the prognosis of muscle‐invasive bladder cancer. It is unclear whether radical cystectomy or systemic pembrolizumab is the optimal next treatment in patients with muscle‐invasive bladder cancer and progressive disease in the pelvic lymph node following neoadjuvant chemotherapy (NAC).

**Case presentation:**

A 62‐year‐old woman with cT3N0M0 bladder cancer and high programmed death‐ligand 1 expression developed solitary obturator lymph node metastasis following 5 cycles of neoadjuvant chemotherapy. Six subsequent cycles of pembrolizumab shrank the lymph node significantly, and conversion radical cystectomy was planned. Pathologically, only carcinoma in situ around the scar of transurethral resection of bladder tumor remained in the primary tumor, and the accumulation of foamy macrophages and fibrosis without viable tumor cells was observed in the dissected lymph node. Eighteen months passed without a recurrence following radical cystectomy.

**Conclusion:**

Pembrolizumab administration before radical cystectomy achieved a good response in a patient with obturator lymph node metastasis following neoadjuvant chemotherapy.

**Abbreviations & Acronyms**
cis: carcinoma in situ
CRP: C‐reactive protein
CT: computed tomography
GC: gemcitabine and cisplatin
Hb: hemoglobin
ICI: immune checkpoint inhibitor
LDH: lactate dehydrogenase
LN: lymph node
LNM: lymph node metastasis
MIBC: muscle‐invasive bladder cancer
MRI: magnetic resonance imaging
NAC: neoadjuvant chemotherapy
NLR: neutrophil lymphocyte ratio
pCR: pathological complete response
PD‐L1: programmed death‐ligand 1
PLND: pelvic lymph node dissection
PLT: platelet
RARC: robot‐assisted radical cystectomy
RC: radical cystectomy
SII: systemic immune‐inflammation index
TURBT: transurethral resection of bladder tumor
WBC: white blood cell
γ‐GTP: γ‐glutamyltranspeptidase

**Keynote message**

It is unclear whether RC or systemic pembrolizumab is the better next treatment in patients with MIBC that has progressed in the pelvic LN following NAC. In such cases, pembrolizumab with a view to converting RC could be an option.

## Introduction

NAC before RC and PLND demonstrated prolonged survival in localized MIBC^1^, ^2^, ^3^ and is recommended as standard treatment.^4^ However, in a minority of cases, MIBC showed pelvic LNM following NAC.^5^ It is unclear whether RC or systemic immunotherapy is more effective for these cases.

Before the advent of ICIs, the prognosis was poor for patients with residual MIBC or LNM after NAC.^6^, ^7^ Recently, perioperative use of ICIs improved the prognosis of MIBC.^8^, ^9^ Adjuvant nivolumab was shown to improve the prognosis of residual MIBC or LNM after NAC in the phase III CheckMate 274 trial.^8^ The effectiveness of neoadjuvant immunotherapy was also reported in the phase II PURE‐01 trial.^9^ However, we could find no reports describing systematic immunotherapy with a view to conversion RC in MIBC patients with pelvic LNM after NAC. Here, we report a patient with cT3N0M0 bladder cancer developing obturator LNM following NAC who markedly responded to pembrolizumab before conversion RC with PLND.

## Case presentation

**Fig. 1:**
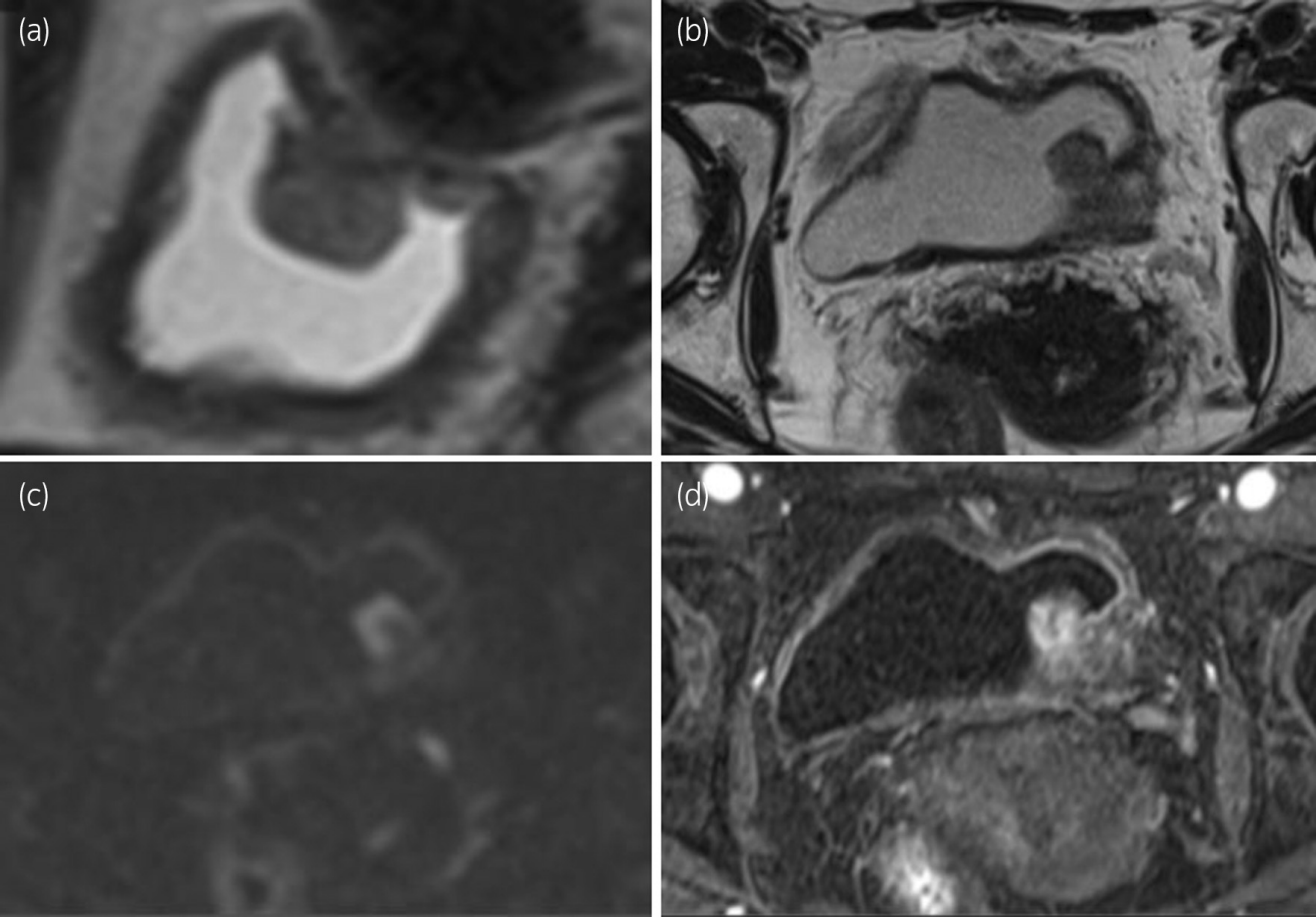
MRI images of cT3N0M0 bladder cancer before TURBT: (a) sagittal view of the T2‐WI image; (b) axial view of the T2‐WI image; (c) axial view of the DWI image; (d) axial view of the contrast‐enhanced image.

**Fig. 2:**
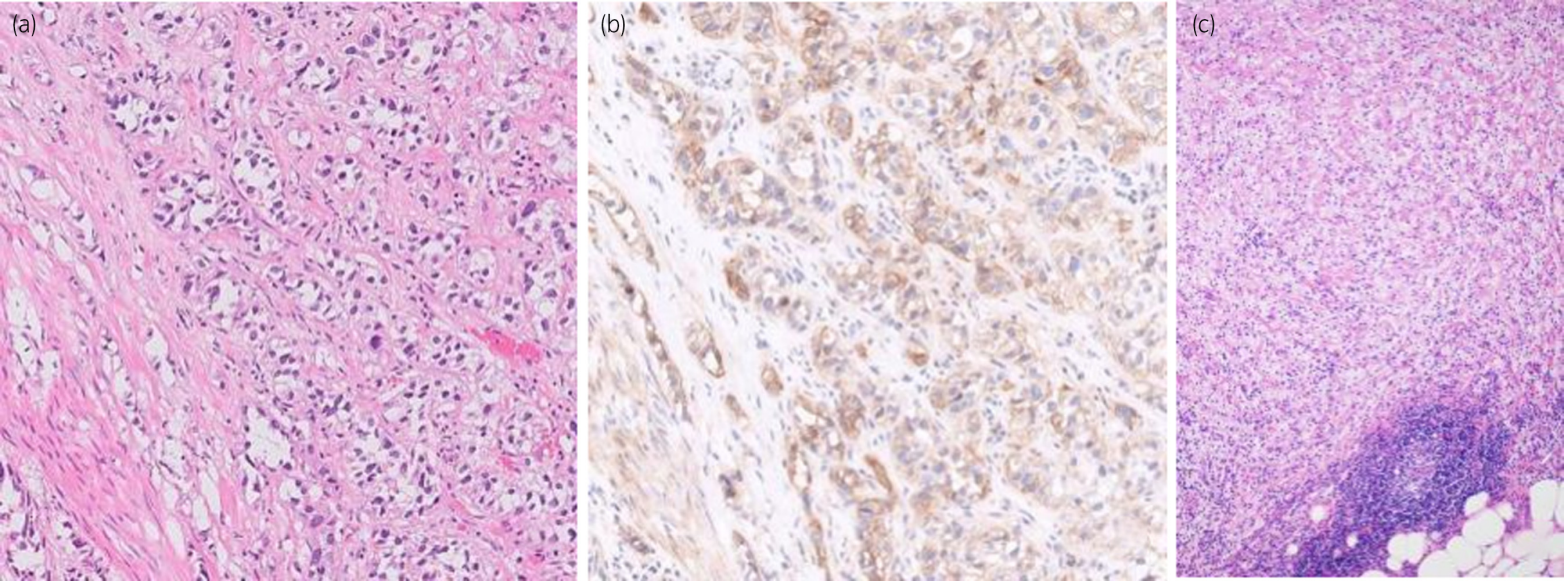
Microscopic examination of the TURBT specimen of the primary tumor showed pT2 and G3 urothelial carcinoma in hematoxylin and eosin staining (a) with PD‐L1 expression in the majority of tumor cells (b). The dissected obturator LN showed an accumulation of foamy macrophages and fibrosis without viable tumor cells (c).

A 62‐year‐old woman visited her doctor because of gross hematuria in 2021. Urine cytology revealed the presence of atypical cells. Cystoscopy and MRI revealed a nodal tumor of VI‐RADS score 2 in the left lateral wall (Fig. 1). TURBT revealed pT2 and G3 urothelial carcinoma with cis (Fig. 2a). Contrast‐enhanced CT showed no metastasis or upper urinary tract tumor. Four cycles of neoadjuvant GC (gemcitabine; 100 mg/m^2^ Days 1, 8, 15, and cisplatin; 70 mg/m^2^ Day 2) every 4 weeks were planned and started at 80% dose; the fourth cycle was reduced to 70% due to myelosuppression.

**Fig. 3:**
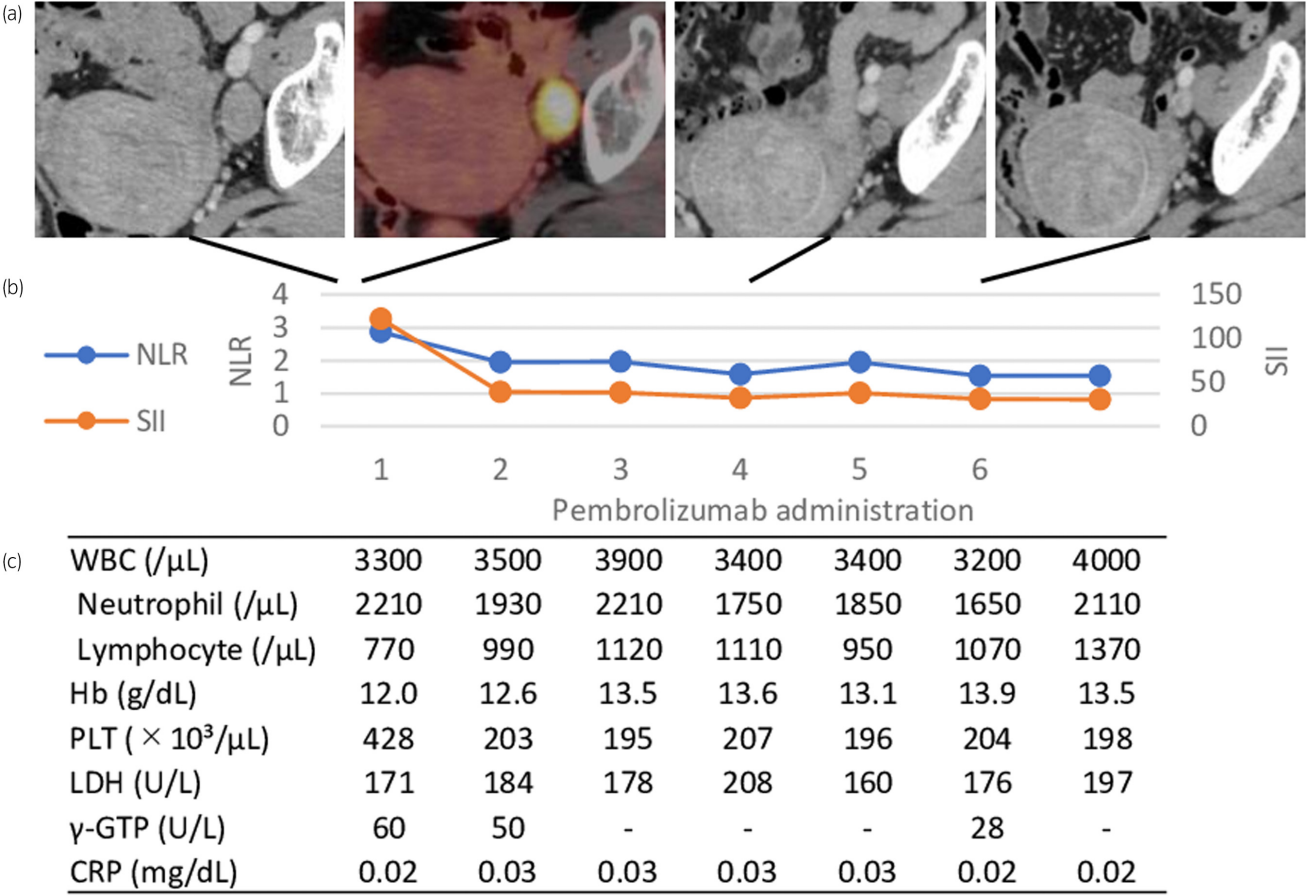
Time‐course of metastatic obturator LN (a) with prognostic factors associated with metastatic urothelial carcinoma (b, c). NLR and SII represent neutrophil/lymphocyte and PLT × neutrophil/lymphocyte, respectively. NLR and SII improved concurrently with the shrinkage of the metastatic LN (b).

The patient was referred to us for RARC. We resumed the fifth cycle of GC at a 70% dose because of the waiting period before RARC. However, after the fifth cycle, a preoperative CT revealed a solitary left obturator LNM (30 mm) with fluorodeoxyglucose uptake in positron emission tomography‐CT (ycT3N1). We considered this case to be associated with a high recurrence rate after RC, and the disease was unable to be resected with curative intent. We began treatment with pembrolizumab 200 mg every 3 weeks with a view to conversion RC in case the LNM shrunk. Three weeks after the first administration of pembrolizumab, the LNM (16 mm) had shrank markedly. After 6 cycles, the LNM had shrunk to an insignificant size, and we planned conversion RC. NLR and SII (NLR × PLT count [×10^3^/μL])^10^ were decreasing concurrently with shrinkage of the LNM (Fig. 3). No immune‐related adverse effects were observed.

RARC with PLND and extracorporeal urinary diversion were performed in 2022. Total operative time and console time were 447 and 272 min, respectively. The estimated blood loss during surgery was 200 mL. Pathologically, only the cis around the scar of TURBT remained in the primary tumor. The dissected obturator LN showed an accumulation of foamy macrophages and fibrosis without viable tumor cells (Fig. 2c), suggesting a good therapeutic effect. Immunohistochemical evaluation using anti‐ PD‐L1 monoclonal antibody (clone 28–8) and the TURBT specimen showed PD‐L1 expression in the majority (60%) of tumor cells (Fig. 2b). No systemic therapy was added following RC. Eighteen months passed without a recurrence.

## Discussion

To the best of our knowledge, there are no reports of preoperative pembrolizumab administration for progressive MIBC following NAC. One report compared pCR to pembrolizumab for chemotherapy‐resistant upper tract urothelial carcinoma.^11^ In the present case, the LNM following neoadjuvant GC disappeared with pembrolizumab, which made it possible to perform conversion RC.

It is unclear whether RC or systemic pembrolizumab is better as the next treatment in MIBC patients with pelvic LNM following NAC. In the former cases, the maximal survival time of 32 ypN+ patients after RC was 3.17 years.^7^ Another study reported that 5‐year overall survival of patients with residual cancer (ypT2‐4 or ypN+) after NAC was 21%, whereas that of cases with pCR was 82%.^12^ Occult LNM after RC also showed a poor prognosis.^13^, ^14^ In the PURE‐01 study of neoadjuvant pembrolizumab, all MIBC patients enrolled in the study underwent RC, and 42% of patients achieved pCR.^9^ Considering the poor prognosis of ypN+ cases after NAC and RC, systemic immunotherapy with a view to converting RC may increase the possibility of cure. In our patient, after pembrolizumab administration, adjuvant nivolumab was covered by Japanese insurance. Subgroup analysis of the CheckMate 274 trial demonstrated the advantage of adjuvant nivolumab in pN+ cases.^8^ The treatment sequence combining RC and systemic immunotherapy for chemotherapy‐resistant cases is an issue yet to be resolved. In the present case, pembrolizumab before RC was effective.

Systemic immunotherapy before RC could lead to further advancement and a missed opportunity for RC. To estimate the efficacy of ICIs, PD‐L1 expression demonstrated predictive value in several clinical trials and meta‐analyses.^8^, ^9^, ^15^, ^16^ Neoadjuvant pembrolizumab achieved pCR in 54.3% of cases with high PD‐L1 expression, compared with 13.3% of cases with low PD‐L1 expression.^9^ In the present case, the primary tumor showed high PD‐L1 expression. Assessment of PD‐L1 expression could be useful in determining the treatment of progressive MIBC after NAC.

A recent risk stratification of metastatic urothelial cancer indicated that NLR, Hb, LDH, and CRP were predictive factors of response to pembrolizumab.^17^, ^18^ SII and γ‐GTP were shown to be prognostic factors for urothelial carcinoma.^10^, ^19^ In the present case, NLR and SII were improving concurrently with the shrinkage of the LNM (Fig. 3). These factors could be useful in assessing the effect of neoadjuvant therapy before conversion surgery.

## Conclusions

Pembrolizumab administration in advance of conversion RC achieved a good response in a patient with obturator LNM after NAC.

## Author contributions

Ichiro Yonese: Conceptualization; investigation; visualization; writing – original draft. Noboru Numao: Conceptualization; investigation; supervision; visualization; writing – review and editing. Kentaro Inamura: Investigation; visualization; writing – review and editing. Yusuke Yoneoka: Writing – review and editing. Ryo Fujiwara: Writing – review and editing. Yosuke Yasuda: Writing – review and editing. Tomohiko Oguchi: Writing – review and editing. Shinya Yamamoto: Writing – review and editing. Takeshi Yuasa: Writing – review and editing. Junji Yonese: Writing – review and editing.

## Conflict of interest

Author T.Y. was supported by grants from MSD.

## Approval of the research protocol by an Institutional Reviewer Board

Not applicable.

## Informed consent

Informed consent was obtained from the patient for publication of this case report.

## Registry and the Registration No. of the study/trial

Not applicable.
